# *Litopenaeus vannamei* BMAL1 Is a Critical Mediator Regulating the Expression of Glucose Transporters and Can Be Suppressed by Constant Darkness

**DOI:** 10.3390/ani11102893

**Published:** 2021-10-04

**Authors:** Lefei Jiao, Tianmeng Dai, Peng Sun, Min Jin, Qicun Zhou

**Affiliations:** Laboratory of Fish Nutrition, School of Marine Sciences, Ningbo University, Ningbo 315211, China; jiaolefei@nbu.edu.cn (L.J.); daitianmeng521@163.com (T.D.); sunpeng@nbu.edu.cn (P.S.); jinmin@nbu.edu.cn (M.J.)

**Keywords:** circadian clock, carbohydrate metabolism, light/dark cycles, crustaceans, aquaculture

## Abstract

**Simple Summary:**

Growing evidence has indicated that glucose absorption exhibits profound circadian rhythmicity, mediated entirely by glucose transporters. We observed that the daily profile of BMAL1, GLUT1 and SGLT1 expression was also synchronized in the intestine and the hepatopancreas of *Litopenaeus vannamei*. Our result identified for the first time that BMAL1 is a critical mediator regulating the expression of glucose transporters, which could be suppressed by constant darkness in *L. vannamei*.

**Abstract:**

Aryl hydrocarbon receptor nuclear translocator-like protein 1 (BMAL1) is a core circadian transcription factor that controls the 24-h cycle of physiological processes. In shrimp, the role of BMAL1 in the regulating glucose metabolism remains unclear. Firstly, we observed that the daily profile of BMAL1, GLUT1 and SGLT1 expression were synchronized in the intestine and the hepatopancreas of *Litopenaeus vannamei*. Then we examined the effects of BMAL1 on the gene expression of glucose transporter type 1 (SGLT1) and sodium-glucose cotransporter 1 (GLUT1) in vivo and in vitro. BMAL1 in *L. vannamei* shares 70.91–96.35% of sequence identities with other shrimp species and possesses the conserved helix-loop-helix domain and polyadenylation site domain. The in vitro dual-luciferase reporter assay and in vivo RNA interference experiment demonstrated that BMAL1 exerted a positive regulation effect on the expression of glucose transporters in *L. vannamei*. Moreover, we conducted an eight-week treatment to investigate whether light/dark cycle change would influence growth performance, and gene expression of BMAL1, GLUT1 and SGLT1 in *L. vannamei*. Our result showed that compared with natural light treatment, constant darkness (24-h darkness) significantly decreased (*p* < 0.05) serum glucose concentration, and suppressed (*p* < 0.05) the gene expression of BMAL1, GLUT1 and SGLT1 in the hepatopancreas and the intestine. Growth performance and survival rate were also decreased (*p* < 0.05) by constant darkness treatment. Our result identified BMAL1 as a critical mediator regulating the expression of glucose transporters, which could be suppressed by constant darkness in *L. vannamei.* It would be quite interesting to explore the mechanism of dark/light cycles on glucose transport and metabolism in *L. vannamei,* which might provide a feeding strategy for improving carbohydrate utilization in the future.

## 1. Introduction

The endogenous circadian clock is a timekeeping system that drives adaptation to physiological and behavioral events based on daily light change in almost all organisms [1]. Studies in animals have suggested that not only can a tissue itself express clock genes, but additionally, numerous biological processes can exhibit circadian rhythmicity, including DNA synthesis, motility and macronutrient absorption [2,3,4]. In crustaceans, the carbohydrate metabolism is under circadian regulation, which can be detected as the rise and fall in glucose levels in the haemolymph of crayfish and crabs [5]. Carbohydrates in the diet are firstly digested to glucose, galactose, and fructose in the small intestine. These sugars are then absorbed by enterocytes lining the upper third of the intestinal villi [6]. It is commonly accepted that glucose is absorbed by transport proteins, such as sodium–glucose linked transporters (SGLTs) and facilitated diffusion glucose transporters (GLUTs), which are embedded in the brush border and basolateral membranes [7]. Growing evidence has indicated that glucose absorption exhibits profound circadian rhythmicity and is mediated entirely by glucose transporters [2,8,9]. However, the regulatory mechanism of circadian clock on glucose transporters has not been well described in crustaceans.

On the molecular level, circadian rhythms are generated by the molecular clock genes that consist of multiple transcriptional/translational negative feedback loops [10]. Among the clock genes, the circadian clock gene aryl hydrocarbon receptor nuclear translocator-like protein 1 (BMAL1) is a core circadian transcription factor that controls the 24-h cycle of physiological processes and systemic deletion of BMAL1 resulted in impaired circadian behavior [11]. Recently, the role of BMAL1 in the regulating glucose metabolism has been identified using gene overexpression and knockout mammals models [12,13,14].Like mammals, aquatic animals also exhibit evident rhythms in physiology behavior and the BMAL1 gene has been identified in a number of fish species [15,16]. However, the role of BMAL1 on the regulation of glucose transporters in crustacean, such as Pacific white shrimp *Litopenaeus vannamei*,remains to be illustrated.

Currently, increasing evidence suggests that change of photoperiod can disrupt circadian rhythms and has disruptive effects on physiology, including growth, immune and cognitive processes, digestion, hormone release, etc [17,18]. This research has been done mainly in mammals. In aquaculture, varied results of different photoperiods on growth and survival have been also observed in the shrimp *Penaeus merguiensis* and *L. vannamei* [19,20]. It has been reported that BMAL1 failed to cycle under constant darkness in Rainbow Trout *Oncorhynchus mykiss* [21]. So far, there have been no reports on whether BMAL1 could regulate glucose transport and whether this influence could be suppressed by photoperiod change in *L. vannamei.*

We hypothesize, based upon these results, that circadian clock gene BMAL1 could regulate glucose transport and this activity could be influenced by photoperiod change in *L. vannamei*. To test this prediction, we firstly examined the effects of BMAL1 on the gene expression of glucose transporter type 1 (GLUT1) and sodium glucose cotransporter 1 (SGLT1) using in vitro gene overexpression (dual-luciferase reporter assay) and in vivo gene knockdown technique (RNA interference). In the last decade, RNA interference (RNAi), a cellular mechanism that uses RNA-guided degradation of messenger RNA transcripts, has had an important impact on identifying and characterizing gene function [22]. A dual-luciferase reporter assay is a common assay in molecular biology that uses the luciferase enzyme and a substrate (such as luciferin) to study whether a protein can activate or repress the expression of a target gene using luciferase as a reporter protein [23]. Secondly, an eight-week feeding trial was designed to investigate whether light/dark cycle change would influence BMAL1 expression, and further influence glucose transport and growth performance in *L. vannamei.*

## 2. Materials and Methods

### 2.1. Gene Expression (BMAL1, GLUT1 and SGLT1) Daily over 24-h in the Intestine and Hepatopancreas

In order to understand the expression of BMAL1, GLUT1 and SGLT1 over 24 h, we sampled shrimp at different time points (6:00, 10:00, 14:00, 18:00, 0:00, and 03:00), with six shrimp at each time point. *L. vannamei* were obtained from Ningbo marine fishery science and technology innovation base and reared in a semi-intensive culture pond with running aerated water at an ambient temperature (28 ± 2 °C) and salinity at 20 ppt. Mid-intestine and hepatopancreas RNA was extracted for RT-qPCR.

### 2.2. Protein Sequence and Domain Structure Analysis

Sequence analysis was performed with the BLAST algorithm (http://www.ncbi.nlm.nih.gov/blast, accessed on 13 July 2021) [24] and the Expert Protein Analysis System (http://www.expasy.org, accessed on 1 October 2020) [25]. Multiple sequence alignment was carried out using DNAMAN and the software GeneDoc. Protein domains were predicted with the simple modular architecture research tool (SMART) version 7.0 (http://smart.embl-heidelberg.de, accessed on 7August 2021) [26].

### 2.3. In Vitro Dual-Luciferase Reporter Assays

The recombinant plasmid pcDNA3.1-BMAL1, GLUT1/SGLT1 firefly plasmid and renilla plasmid were co-transfected into HEK293T cells and cultured in 24-well plates. There plasmids were constructed by Hangzhou Biospring Co., Ltd (Hangzhou, China). At 48 h post-transfection, renilla luciferase activities were detected by using the dual-luciferase reporter assay system (Promega, Madison, Mis, USA) according to the manufacturer’s instructions. The plasmid pcDNA3.1-BMAL1 was used as the control group. This experiment was performed in three independent transfections.

### 2.4. In Vivo Knockdown by dsRNA Injection

Interference sites were designed at the 5 end (400-500bp) of the ORF region of the BMAL1 gene. The dsRNA-specific targets of BMAL1 and green fluorescent protein (GFP) were synthesized (Table 1) in vitro using the T7 RiboMAX Express kit (Promega, USA). A total of 28 shrimp (an average weight of 8.10g) were divided into 2 groups (14 in each group): Negative control group (GFP dsRNA injection) and BMAL1 dsRNA interference group (injection of BMAL1-specific dsRNA). DsRNA was injected into the shrimp with a 1 mL syringe through the connecting part of the shrimps cheliped. Each shrimp was injected with 10 μg dsRNA, and six shrimp in each group were sampled at 48 h post-injection. Tissue RNA (mid-intestine and hepatopancreas) was extracted for RT-qPCR.

### 2.5. Eight-Week Light/Dark Treatment

The shrimp were randomly divided into two groups: the natural light (12 h light/12 h dark) and dark treatment (24 h dark) groups, with four replicates per group. A total of 240 shrimp with an average weight of 0.72 g were randomly assigned into eight 100-L cylindrical fiberglass tanks with 30 shrimp per tank. Shrimp were fed with pellet feed (containing 45.71% crude protein, 7.36% crude lipid) which was produced in our lab. After eight weeks, shrimp in each tank were counted and final body weight (FBW) was taken to determine the percent weight gain (PWG) and survival rate. The intestinal segment (mid-intestine) and hepatopancreas of ten shrimp in each tank were harvested, mixed as one replicate, rapidly frozen in liquid nitrogen, and stored at – 80 °C for further PCR analysis. Hemolymph samples of ten shrimp in each tank were collected from the pericardial cavity and placed into 1.5 mL centrifuge tubes overnight at 4 °C before centrifugation (4000 rpm, 10 min). The supernatant was mixed as one replicate and collected for analysis of glucose levels. Glucose levels were determined using Selectra ProM Clinical Chemistry System (ELITech, Sees, France).

### 2.6. Quantitative Reverse Transcription PCR (RT-qPCR)

The total RNA was extracted from samples using TRIzol reagent (Vazyme Biotech Co., Ltd., Nanjing, China), and its quality was subsequently measured using a Nano-Drop ND-2000 spectrophotometer (Nano-Drop Technologies, Wilmington, DE, USA). The cDNA was synthesized using the Primer-Script One Step RT-PCR Kit (Vazyme Biotech Co., Ltd., Nanjing, China). All the primer sequences were designed using the Primer Premier 5.0 software and are listed in Table 1. *β-actin* was used as a housekeeping gene. The relative expression levels of genes (BMAL1, GLUT1 and SGLT1) were determined by RT-qPCR using a SYBR Premix Ex TaqTM kit (Vazyme Biotech co., Ltd). The amplification program for RT-qPCR was as follows: 95 °C for 2 min, followed by 45 cycles of 95 °C for 10 s, 58 °C for 10 s, and 72 °C for 20 s. The 2^−ΔΔCt^ method was used to analyze the relative expression (fold changes) of target genes. All samples were run in triplicate.

### 2.7. Statistical Analysis

An independent-sample t test was used to compare the data using the SPSS 20.0 statistical package (SPSS Inc., Chicago, IL, USA). The data were expressed as mean ± standard deviation (SD). *p* < 0.05, *p* < 0.01 and *p* < 0.001 were considered statistically significant.

## 3. Results

### 3.1. Daily Profile of BMAL1, GLUT1 and SGLT1 Expression in the Hepatopancreas and Intestine

**Figure 1:**
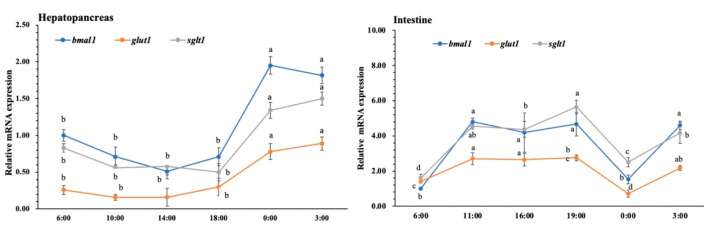
Daily profile of BMAL1,GLUT1 and SGLT1 expression in the intestine and hepatopancreas. Six shrimp were sampled at each time point (n = 6). Data presented are mean ± SD, and the different letters above the broken line represent significant differences between different time points (*p* < 0.05).

Figure 1 shows the daily profile of BMAL1, GLUT1 and SGLT1 expression in the hepatopancreas and intestine of *L. vannamei.* We found that the daily profile of BMAL1, GLUT1 and SGLT1 expression was synchronized in intestine and hepatopancreas. The expression of BMAL1, GLUT1 and SGLT1 reached the high value (*p* < 0.05) at 0:00 and 3:00 in the hepatopancreas compared with other time points. In the intestine, the expression of BMAL1, GLUT1 and SGLT1 increased (*p* < 0.05) after 6:00 and then decreased (*p* < 0.05) at 0:00.

### 3.2. The Sequence Characteristics of BMAL1

**Figure 2:**
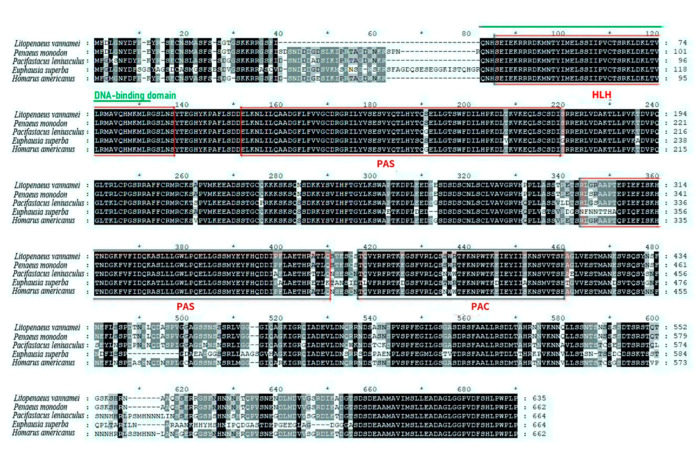
Alignment of the sequences of BMAL1. The consensus residues are in black, the conserved domains and motifs are indicated in red, and DNA-binding domains are indicted in green. The genBank accession numbers of the aligned sequences are as follows: *L. vannamei*, ROT67541.1; *Pa. leniusculus*, AFV39705.1; *E. superba*, ANW48377.1; *H. americanus*, AWC08576.1; *Pe. monodon*, XP_037792710.1.

As shown in Figure 2, the predicted BMAL1 protein sequence contains 635 amino acid residues with a molecular mass of 70.32 kDa. It contains a helix-loop-helix (HLH) domain (residues 40–91), two conserved polyadenylation site (PAS) domains (residues 106–173 and 298–364), and a motif C-terminal to PAS (PAC, residues 371–414) (Figure 1). Within the HLH domain is a DNA binding region (residues 70-152) that contains an E-box/N-box specificity site. *L. vannamei* BMAL1 shares 70.91–96.35% of sequence identities with other shrimp species, including *Euphausia superba*, *Pacifastacus leniusculus*, *Homarus americanus* and *Pe. monodon.*

### 3.3. Overexpression of BMAL1 Significantly Activated the Gene Expression of GLUT1 and SGLT1 In Vitro

**Figure 3:**
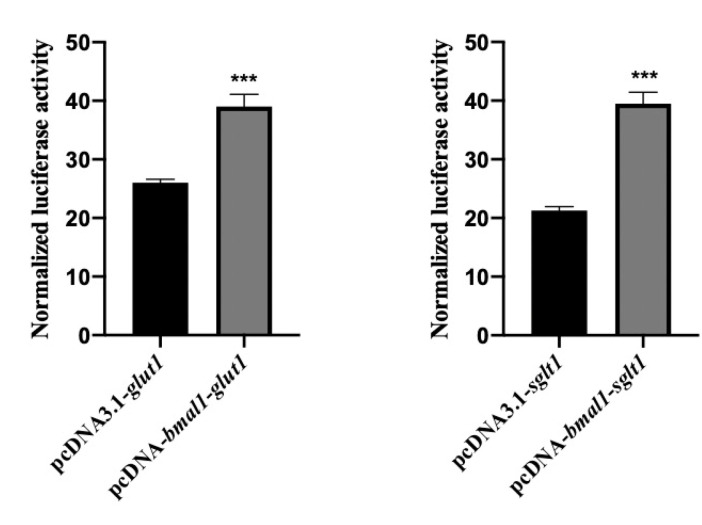
Overexpression of BMAL1 in vitro significantly activated the expression of GLUT1 and SGLT1 in *L. vannamei*. Each bar represents the mean ± SD (n = 4). Bars assigned with asterisks are significantly different. *** indicated *p* < 0.001.

As shown in Figure 3, luciferase activities in the pcDNA3.1-BMAL1-GLUT1 group were significantly upregulated (*p* < 0.001) compared to those in the control pcDNA3.1-GLUT1 group. Similarly, luciferase activities in the pcDNA3.1-BMAL1-SGLT1 group were significantly upregulated (*p* < 0.001) compared to those in the control pcDNA3.1-SGLT1 group.

### 3.4. Knockdown of BMAL1 Decreased the mRNA Expression of Glucose Transporters (GLUT1, SGLT1) in the Hepatopancreas and Intestine of L. vannamei

**Figure 4:**
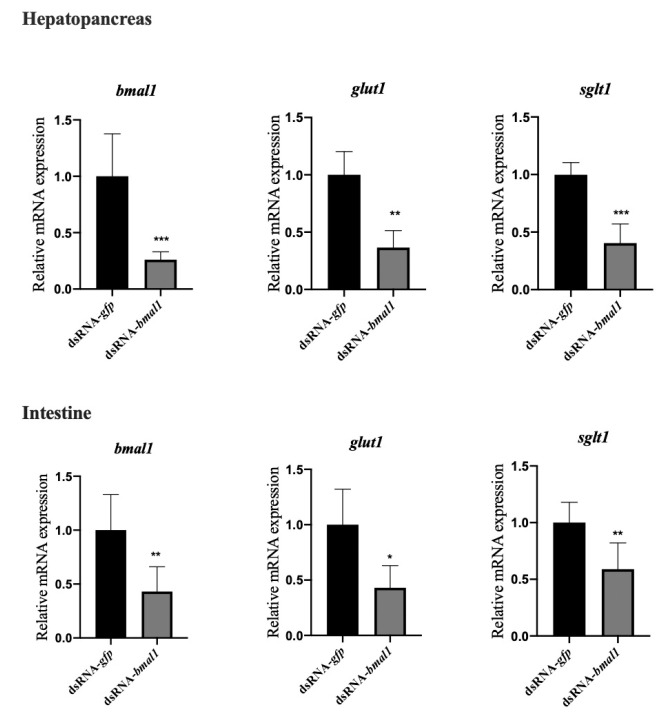
The knockdown of BMAL1 decreased the mRNA expression of glucose transporters (GLUT1, SGLT1) in the hepatopancreas and intestine of *L. vannamei.* Each bar represents the mean ± SD of six samples. Bars assigned with asterisk are significantly different. * indicates *p* < 0.05, ** indicates *p* < 0.01, indicates *** *p* < 0.001).

To further confirm that BMAL1 could regulate glucose transport by regulating the mRNA expression of GLUT1 and SGLT1, the in vivo knockdown of BMAL1 was performed based on double-stranded RNA interference. As shown in Figure 4, down-regulated BMAL1 expression in the dsRNA-BMAL1 group confirmed the successful interference of BMAL1. After the silencing of BMAL1, the mRNA levels of GLUT1 and SGLT1 in the hepatopancreas and intestine were significantly lower (p < 0.05) than those in the dsRNA-GFP group, suggesting that BMAL1 could positively regulate the mRNA expression of GLUT1 and SGLT1.

### 3.5. Effects of Dark Treatment on Growth Performance and Survival Rate in L. vannamei

**Figure 5:**
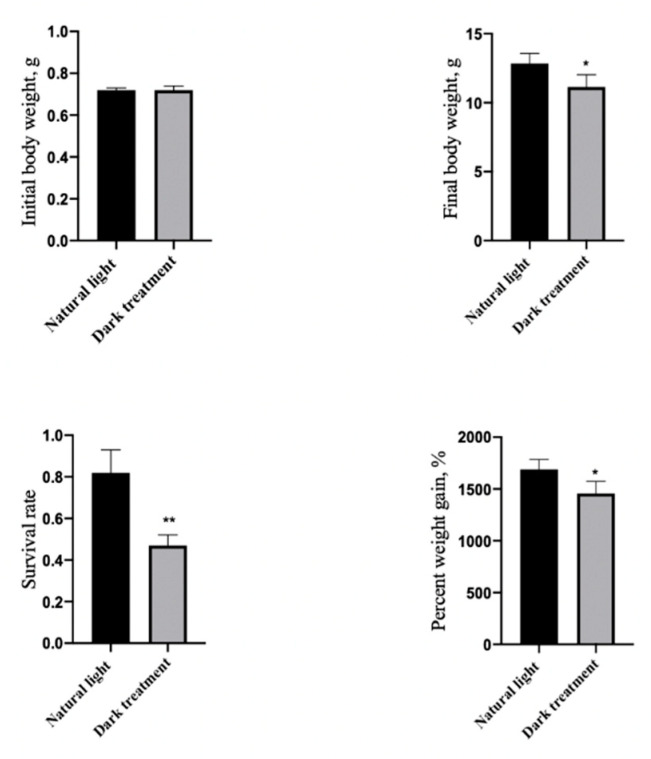
Effects of dark treatment on growth performance in *L. vannamei.* Values are represented as mean ± SD (n = 4) by vertical bars. Bars assigned with asterisks are significantly different (* indicates *p* < 0.05, ** indicates *p* < 0.01).

As Figure 5 shows, compared with the control group (natural light), dark treatment significantly decreased (*p* < 0.05) the FBW, PWG, and survival rate in *L. vannamei.*

### 3.6. Effects of Dark Treatment on Serum Glucose Concentration in L. vannamei

**Figure 6:**
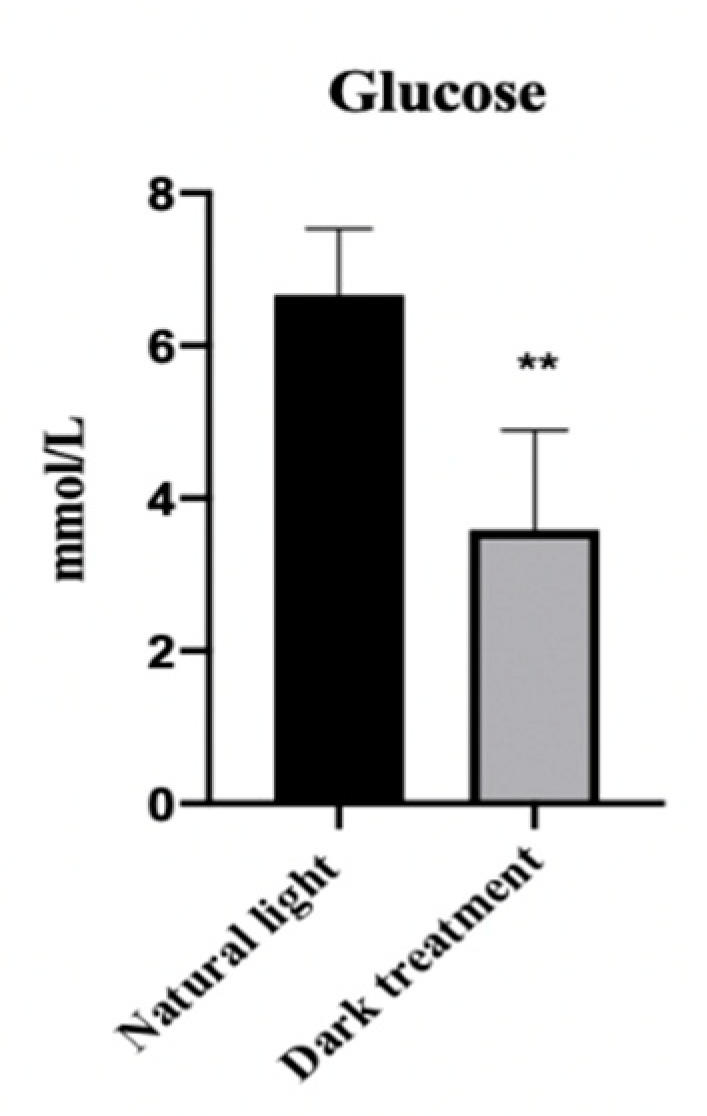
Effects of dark treatment on serum glucose concentration in *L. vannamei.* Values are represented as mean ± SD (n = 4) by vertical bars. Bars assigned with asterisk ** are significantly different (*p* < 0.01).

As Figure 6 shows, compared with the control group (natural light), dark treatment significantly decreased (*p* < 0.05) the serum glucose concentration in *L. vannamei.*

### 3.7. Effects of Dark Treatment on the mRNA Expression of BMAL1, GLUT1 and SGLT1 in the Hepatopancreas and Intestine of L. vannamei

**Figure 7:**
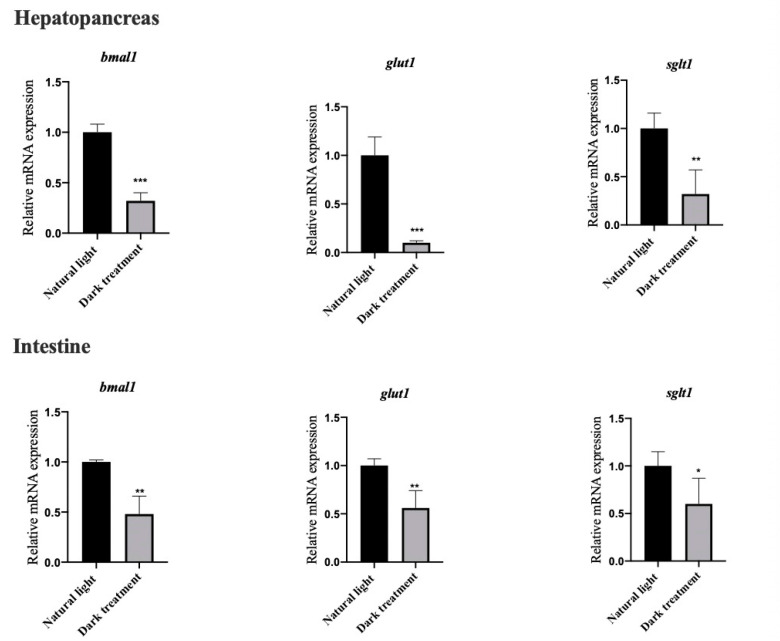
Effects of dark treatment on the mRNA expression of BMAL1, GLUT1 and SGLT1 in the hepatopancreas and intestine of *L. vannamei.* Values are represented as mean ± SD (n = 4) by vertical bars. Bars assigned with asterisks are significantly different (* indicates *p* < 0.05, ** indicates *p* < 0.01, *** indicates *p* < 0.001).

Figure 7 shows that eight weeks of dark treatment significantly decreased (*p* < 0.05) the mRNA expression of BMAL1, GLUT1 and SGLT1 in the hepatopancreas and intestine of *L. vannamei.*

## 4. Discussion

It is now emerging that circadian clocks bring about rhythmic changes in downstream molecular pathways and physiological processes such as nutrition metabolism [27,28]. Currently, the role of circadian clock genes in the rhythmicity of glucose absorption is not well understood in aquatic animals. Cellular uptake of glucose is mediated by two different types of membrane associated carrier proteins, including SGLTs and GLUTs [29]. In the present study, we observed that the daily profile of BMAL1, GLUT1 and SGLT1 expression was synchronized in the intestine and hepatopancreas. After this, dual-luciferase reporter assays confirmed that BMAL1 and glucose transporter genes (GLUT1 and SGLT1) interacted. We further conducted the RNA interference experiment to confirm the mechanism of interaction between BMAL1 and glucose transporter genes (GLUT1 and SGLT1) at mRNA level. BMAL1 knockdown decreased the mRNA expression of GLUT1 and SGLT1 in the hepatopancreas and intestine of *L. vannamei,* presenting convincing evidence that BMAL1 contributed to glucose uptake *in vivo*. Similarly, inactivation of BMAL1 suppressed the diurnal variation in glucose and abolished gluconeogenesis in mice [14]. BMAL1 regulated glucose uptake in differentiated intestinal epithelial cells (caco-2) and was dependent on the glucose transporter SGLT1 [13]. Further mechanism study demonstrated that BMAL1 knockdown decreased glucose uptake by inhibiting the NAMPT/NAD+/SIRT1 pathway and GLUT4 expression in HepG2 cells [30]. The basic helix-loop-helix (b-HLH)-PAS proteins CLOCK and BMAL1 are core elements of this system and function as transcriptional activators to drive the expression of multiple clock-controlled genes [31]. Based on our result, the glucose transporters might be some of these targets in *L. vannamei*. However, it has to be acknowledged that for many proteins transcription and translation are not tied. Thus, to show unequivocally that BMAL1 can regulate glucose transporters, studies at protein level need to be done.

In aquatic farming, abiotic factors such as photoperiod are believed to play an important role in promising successful grow-out period with good survival and good growth rate. Up to now, research has investigated the effects of photoperiod on larval survival and growth in several species of crustacean and the results varied with species, experiment design, growth stage, etc. Marine blue swimmer crab *Portunus. pelagicus* showed no significant difference in survival from zoea to megalopa stage under different photoperiod regimes [32]. Hoang et al. (2003) stated that photoperiod imposing two light/dark cycles (7 h light/5 h dark) in 24 h resulted in a significantly higher growth of *P.merguinses* juveniles than the normal photoperiod (12 h light/12 h dark) [20]. Sanudin et al., (2014) found that *L. vannamei* (initial length 0.5 cm) under dark conditions decreased the ingestion rates of frozen *Artemia nauplii,* but had no influence on growth and survival rate for three weeks [19]. In the present study, our results demonstrated that constant darkness decreased the growth performance and survival rate in *L. vannamei* under eight-week light/dark cycle treatment. Differences in photoperiod, feed, and breeding cycle might result in inconsistencies in the results.

As a crucial environmental factor, photoperiod plays an important role in many behavioural and physiological functions in living organisms [33]. Animal studies demonstrate that constant light/dark exposure could disrupt many aspects of circadian rhythm and has disruptive effects on metabolic processes [34]. Light influence is a growing concern to the biology and ecology of exposed marine organisms, such as zebrafish, coral reef fish and *Sparidae. aurata* [35,36,37]. However, the adverse effects of disturbed dark/light rhythm are not fully understood for invertebrates [38,39]. In recent years, the role of photoperiod in modulating the expression of circadian clock protein is receiving more attention [40]. Aberrant expression of circadian clock genes has been observed after constant light/darkness treatment. In the present study, our data demonstrated for the first time that constant darkness could suppress the mRNA expression of BMAL1 in *L. vannamei.* Similarly, both constant light or darkness resulted in dramatically decreased BMAL1 mRNA expression at zeitgeber time (ZT) 0 and ZT4, and decreased total BMAL1 mRNA and protein expression in the brain and muscle of rats [30]. Meanwhile, the mRNA expression of tissue (hepatopancreas and intestine) glucose transporters (GLUT1 and SGLT1) and serum glucose levels were decreased in *L. vannamei.* The decreased BMAL1 mRNA expression might partially explain the decreased ability of glucose absorption in *L. vannamei*. In addition, levels of crustacean hyperglycaemic hormones undergo circadian changes and might participate in the regulation of glucose homeostasis since a temporary loss of synchrony occurred in constant darkness [4,41]. In mouse models, circadian misalignment induced by constant darkness could lead to insulin resistance [42]. Taken together, these results provide a new understanding of the mechanisms involved in adverse effects of dark condition on the nutritional physiology in *L. vannamei*. It is well-known that crustaceans have a limited ability to utilize carbohydrates and cannot adapt to high levels of dietary carbohydrates [43]. Compared with those in fish, few studies have examined the nutrition or metabolism of crustacean species. This lack of information is a bottleneck for current understanding of crustacean physiology. Therefore, it would be quite interesting to explore the mechanism of dark/light cycles on glucose transport and metabolism in *L. vannamei*, which might provide a potential feeding strategy (changing photoperiod) for improving carbohydrate utilization in the future.

## 5. Conclusions

In conclusion, in vitro and in vivo experiments identified that BMAL1 exerted a positive regulation effect on the expression of glucose transporters in *L. vannamei*. Moreover, an eight-week light/dark cycle treatment showed that constant darkness could decrease growth performance and suppress BMAL1 and glucose transport expression in *L. vannamei.* It would be quite interesting to explore the mechanism of dark/light cycles on glucose transport and metabolism in *L. vannamei*, which might provide a potential feeding strategy for improving carbohydrate utilization in the future.

## Figures and Tables

**Table 1:** Summary of primers used in this study.

Primers	Sequences (5’ to 3’)
For RNA interference experiment	
GFP-F	ATGGTGAGCAAGGGCGAGGAG
GFP-R	TTACTTGTACAGCTCGTCCATGCC
T7-GFP-F	GGATCCTAATACGACTCACTATAGGATGGTGAGCAAGGGCGAGGAG
T7-GFP-R	GGATCCTAATACGACTCACTATAGGTTACTTGTACAGCTCGTCCATGCC
BMAL1-F	ATGTTTGATCTTGGCAACTATG
BMAL1-R	AAAGTTTGATACACCGATTCCG
T7-BMAL1-F	GGATCCTAATACGACTCACTATAGGATGTTTGATCTTGGCAACTATG
T7-BMAL1-R	GGATCCTAATACGACTCACTATAGGAAAGTTTGATACACCGATTCCG
For RT-PCR	
BMAL1-F	CCTTCAACCACAATAACAACCATAC
BMAL1-R	CCTCGTCTGAGTCGCTCGTGCCATC
GLUT1-F	CTTGGAGTTGGGTCGGTGATGGTTA
GLUT1-R	TCCACGGAATACTGCCAGGACCCAC
SGLT1-F	ATCGGCTTGGTCATAGGACTCATTC
SGLT1-R	GCAGCCGAAGTGGAGATAATGGACA
β-actin-F	CCACGAGACCACCTACAAC
β-actin-R	AGCGAGGGCAGTGATTTC

## Data Availability

The data presented in this study are available on request from the corresponding author.
